# Supplementary material for: Fertility correlates with queen size and sperm quality in an ant
Source: PLoS One. 2025 Dec 10;20(12):e0336378. doi: 10.1371/journal.pone.0336378 (PMC12694860; doi:10.1371/journal.pone.0336378)
Supplement: S1 File — (DOCX) [file pone.0336378.s001.docx]

**Supplementary Figures**


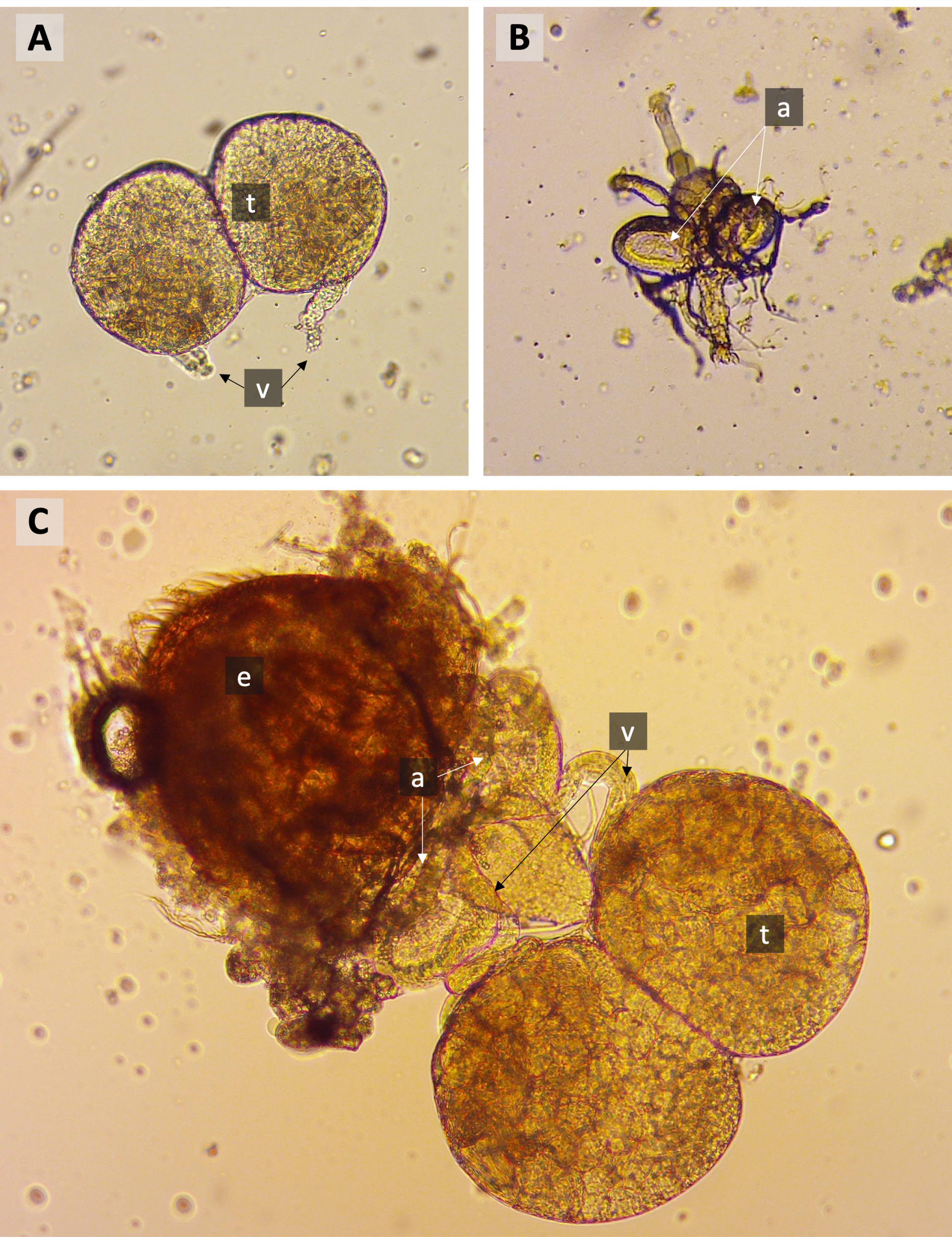


**Supplementary Figure 1. Dissection of the internal reproductive organs of wingless males in *Cardiocondyla obscurior*.** A): Testes (t) with attached paired seminal vesicles (v). B): Detailed microscopy of paired accessory glands (a), separated from the testes and seminal vesicles. C): Overall morphology of the reproductive tract of wingless males with (a) accessory glands, (t) testes, (v) seminal vesicles, and (e) external genitalia.

**Supplementary Figure 2. Queen size and queen pupae production.** No significant correlation was found between the queen thorax length and the number of queen pupae produced.


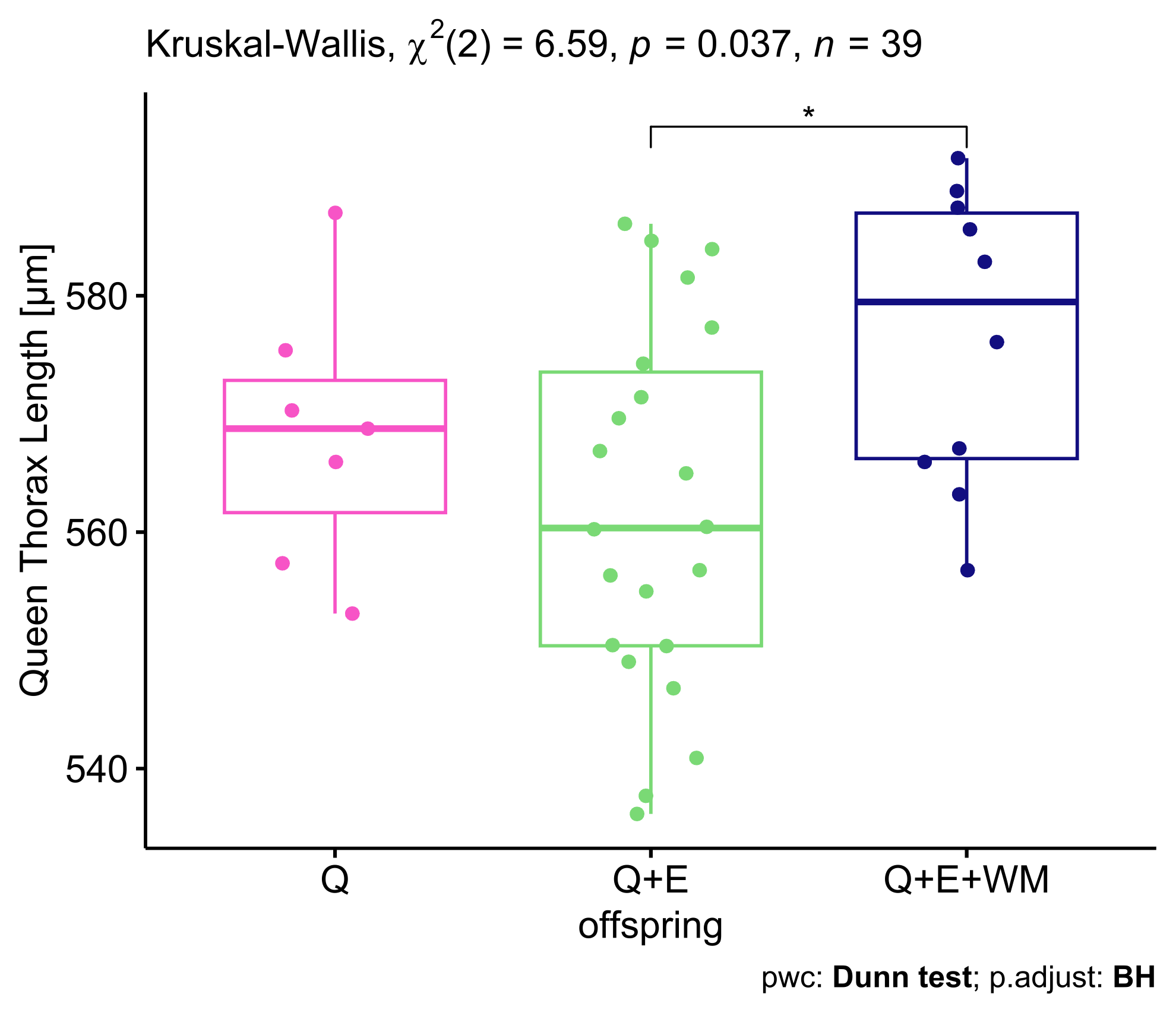


**Supplementary Figure 3. Relation between queen size and composition of sexual offspring.** The thorax length of the queens plotted against the different groups of sexual offspring produced. A Kruskal-Wallis test was performed, and post-hoc pairwise comparisons were made using Dunn's test with BH adjustement. Queens that produced female sexuals and wingless (ergatoid) males (nQ+E = 22) were significantly smaller compared to queens that also produced winged male offspring (nQ+E+WM = 10, p = 0.033). No difference was found comparing those groups to queens that only produced queen offspring (nQ+E = 22, p = 0.318; nQ+E+WM = 10, p = 0.318). Boxplots show medians, 25 and 75 quartiles, and 95% percentiles.


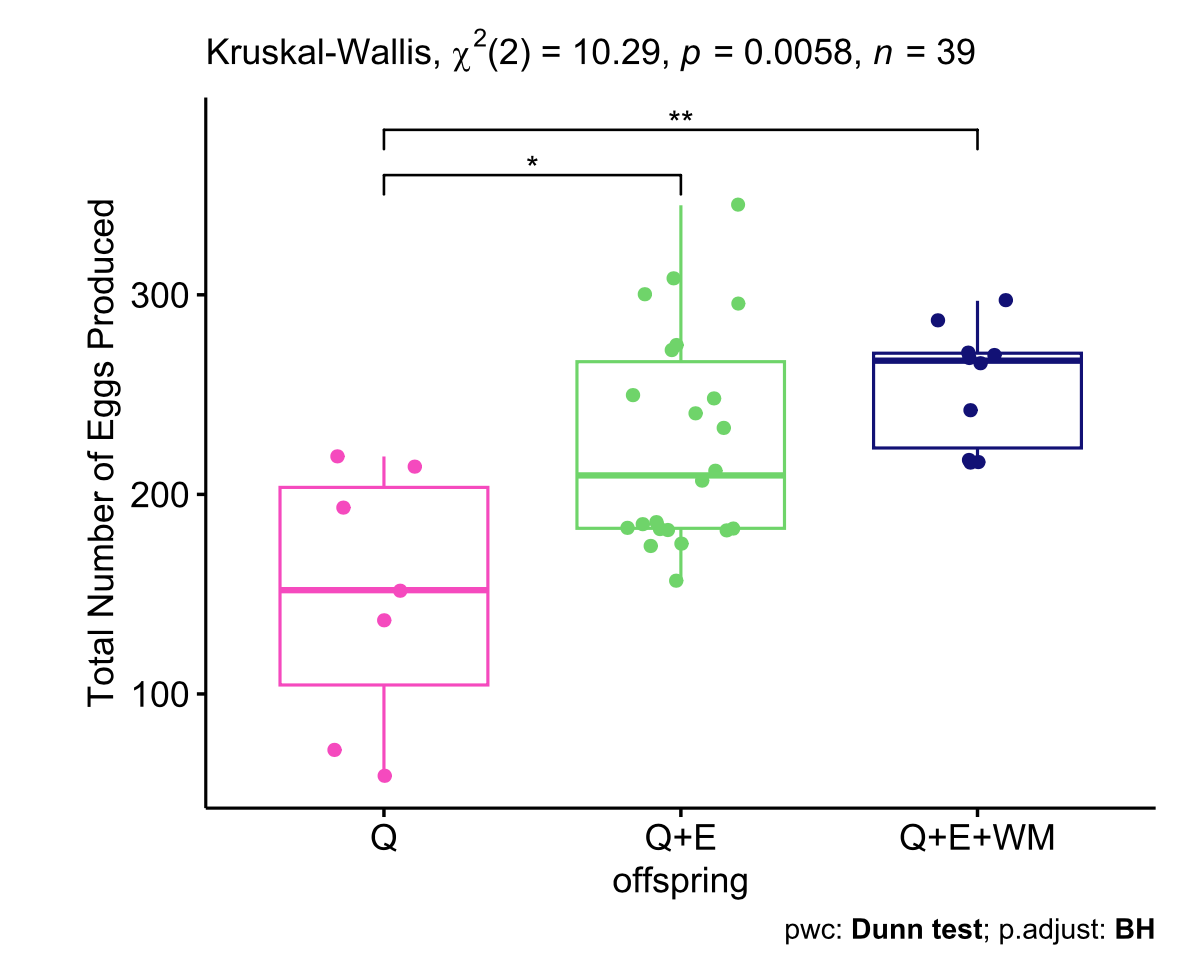


**Supplementary Figure 4. Relation between total egg production and composition of sexual offspring.** The total egg production of the queens plotted against the different groups of sexual offspring produced. A Kruskal-Wallis test was performed, and post-hoc pairwise comparisons were made using Dunn's test with BH adjustment. Queens that only produced female sexuals (nQ = 7) laid significantly fewer eggs than queens producing also ergatoid males (nQ+E = 22; p = 0.043) and winged male offspring (nQ+E+WM = 10, p = 0.004). No difference was found comparing queens with male offspring (nQ+E = 22 and nQ+E+WM = 10, p = 0.097). Boxplots show medians, 25 and 75 quartiles, and 95% percentiles.
