# Supplementary material for: Fertility correlates with queen size and sperm quality in an ant
Source: PLoS One. 2025 Dec 10;20(12):e0336378. doi: 10.1371/journal.pone.0336378 (PMC12694860; doi:10.1371/journal.pone.0336378)
Supplement: Attachment — Submitted filename: PONE-D-25-42359_reviewer.pdf [file pone.0336378.s004.pdf]

PLOS ONE

Fertility correlates with queen size and sperm quality in an ant

--Manuscript Draft--

**Manuscript Number:**	PONE-D-25-42359
**Article Type:**	Research Article
**Full Title:**	Fertility correlates with queen size and sperm quality in an ant
**Short Title:**	Ant queen fertility
**Corresponding Author:**	Luisa Maria Jaimes-Nino<br>University of Regensburg Faculty of Biology and Pre-Clinical Medicine: Universitat Regensburg Fakultat fur Biologie und Vorklinische Medizin<br>GERMANY
**Keywords:**	caste ratio; fecundity; sex ratio; social insects; sperm traits
**Abstract:**	Fitness can vary between individuals of the same population for reasons related to nutrient acquisition during development, but also to the quality of the mating partners. We focus on ant queens of Cardiocondyla obscurior to investigate the effects of female and male morphology and sperm characteristics on productivity. We monitored queens for 12 weeks after mating as a proxy for lifetime productivity, and found that larger queens are more productive, as commonly found in other insects. Sperm viability, but not sperm length, was positively correlated with female-biased sex ratios, pointing to a better insemination rate. The high correlation between sperm viability, male body size and mandible size suggests strong selection for larger and stronger males. However, male competition for access to unmated queens in the maternal colony may result in a trade-off between male size and developmental time in this species.
**Order of Authors:**	Lena-Marie Süß
	Jan Oettler
	Luisa Maria Jaimes-Nino
**Additional Information:**	
**Question**	**Response**
**Financial Disclosure**<br><br>Enter a financial disclosure statement that describes the sources of funding for the work included in this submission. Review the submission guidelines for detailed requirements. View published research articles from *PLOS ONE* for specific examples.<br><br>This statement is required for submission and **will appear in the published article** if the submission is accepted. Please make sure it is accurate.	Yes

Powered by Editorial Manager® and ProduXion Manager® from Aries Systems Corporation

**Funded studies**<br>Enter a statement with the following details:<br>• Initials of the authors who received each award<br>• Grant numbers awarded to each author<br>• The full name of each funder<br>• URL of each funder website<br>• Did the sponsors or funders play any role in the study design, data collection and analysis, decision to publish, or preparation of the manuscript?<br><br>Did you receive funding for this work?	
Please add funding details.<br>as follow-up to "**Financial Disclosure**<br><br>Enter a financial disclosure statement that describes the sources of funding for the work included in this submission. Review the submission guidelines for detailed requirements. View published research articles from *PLOS ONE* for specific examples.<br><br>This statement is required for submission and **will appear in the published article** if the submission is accepted. Please make sure it is accurate.<br><br>**Funded studies**<br>Enter a statement with the following details:<br>• Initials of the authors who received each award<br>• Grant numbers awarded to each author<br>• The full name of each funder<br>• URL of each funder website<br>• Did the sponsors or funders play any role in the study design, data collection and analysis, decision to publish, or preparation of the manuscript?<br><br>Did you receive funding for this work?"	JO: OE549/2-2,3 Deutsche Forschungsgemeinschaft https://www.dfg.de/<br>LMJN: JA3614/1-1 Deutsche Forschungsgemeinschaft https://www.dfg.de/<br>FAS-G to LMJN Finanzielles Anreizsystem zur Förderung der Gleichstellung of the University of Regensburg https://www.uni-regensburg.de/humanwissenschaften/frauenbeauftragte/frauenfoerderung/index.html<br><br>No funding sources were involved in study design, data collection and interpretation, or the decision to submit the work for publication.
Please select the country of your main research funder (please select carefully as in some cases this is used in fee calculation).	GERMANY - DE

as follow-up to "**Financial Disclosure**<br><br>Enter a financial disclosure statement that describes the sources of funding for the work included in this submission. Review the submission guidelines for detailed requirements. View published research articles from *PLOS ONE* for specific examples.<br><br>This statement is required for submission and **will appear in the published article** if the submission is accepted. Please make sure it is accurate.<br><br>**Funded studies**<br>Enter a statement with the following details:<br>• Initials of the authors who received each award<br>• Grant numbers awarded to each author<br>• The full name of each funder<br>• URL of each funder website<br>• Did the sponsors or funders play any role in the study design, data collection and analysis, decision to publish, or preparation of the manuscript?<br><br>Did you receive funding for this work?"	
**Competing Interests**<br><br>Use the instructions below to enter a competing interest statement for this submission. On behalf of all authors, disclose any competing interests that could be perceived to bias this work—acknowledging all financial support and any other relevant financial or non-financial competing interests.<br><br>This statement is required for submission and **will appear in the published article** if the submission is accepted. Please make sure it is accurate and that any funding sources listed in your Funding Information later in the submission form are also declared in your Financial Disclosure statement.	The authors have declared that no competing interests exist.

Powered by Editorial Manager® and ProduXion Manager® from Aries Systems Corporation

View published research articles from *PLOS ONE* for specific examples.<br><br>**NO authors have competing interests**<br><br>Enter: *The authors have declared that no competing interests exist.*<br><br>**Authors with competing interests**<br><br>Enter competing interest details beginning with this statement:<br><br>*I have read the journal's policy and the authors of this manuscript have the following competing interests: [insert competing interests here]*<br><br>* typeset	
**Ethics Statement**<br><br>Enter an ethics statement for this submission. This statement is required if the study involved:<br><br>• Human participants<br>• Human specimens or tissue<br>• Vertebrate animals or cephalopods<br>• Vertebrate embryos or tissues<br>• Field research<br><br>Write "N/A" if the submission does not require an ethics statement.<br><br>General guidance is provided below. Consult the submission guidelines for detailed instructions. **Make sure that all information entered here is included in the Methods section of the manuscript.**	N/A

Powered by Editorial Manager® and ProduXion Manager® from Aries Systems Corporation

**Format for specific study types**<br><br>**Human Subject Research (involving human participants and/or tissue)**<br>• Give the name of the institutional review board or ethics committee that approved the study<br>• Include the approval number and/or a statement indicating approval of this research<br>• Indicate the form of consent obtained (written/oral) or the reason that consent was not obtained (e.g. the data were analyzed anonymously)<br><br>**Animal Research (involving vertebrate animals, embryos or tissues)**<br>• Provide the name of the Institutional Animal Care and Use Committee (IACUC) or other relevant ethics board that reviewed the study protocol, and indicate whether they approved this research or granted a formal waiver of ethical approval<br>• Include an approval number if one was obtained<br>• If the study involved *non-human primates*, add *additional details* about animal welfare and steps taken to ameliorate suffering<br>• If anesthesia, euthanasia, or any kind of animal sacrifice is part of the study, include briefly which substances and/or methods were applied<br><br>**Field Research**<br><br>Include the following details if this study involves the collection of plant, animal, or other materials from a natural setting:<br>• Field permit number<br>• Name of the institution or relevant body that granted permission	
**Data Availability**<br><br>Authors are required to make all data underlying the findings described fully available, without restriction, and from the time of publication. PLOS allows rare exceptions to address legal and ethical concerns. See the PLOS Data Policy and FAQ for detailed information.	Yes - all data are fully available without restriction

A Data Availability Statement describing where the data can be found is required at submission. Your answers to this question constitute the Data Availability Statement and **will be published in the article**, if accepted.<br><br>**Important:** Stating 'data available on request from the author' is not sufficient. If your data are only available upon request, select 'No' for the first question and explain your exceptional situation in the text box.<br><br>Do the authors confirm that all data underlying the findings described in their manuscript are fully available without restriction?	
**Describe where the data may be found in full sentences. If you are copying our sample text, replace any instances of XXX with the appropriate details.**<br><br>• If the data are **held or will be held in a public repository**, include URLs, accession numbers or DOIs. If this information will only be available after acceptance, indicate this by ticking the box below. For example: *All XXX files are available from the XXX database (accession number(s) XXX, XXX.)*.<br>• If the data are all contained **within the manuscript and/or Supporting Information files**, enter the following: *All relevant data are within the manuscript and its Supporting Information files.*<br>• If neither of these applies but you are able to provide **details of access elsewhere**, with or without limitations, please do so. For example:<br><br>*Data cannot be shared publicly because of [XXX]. Data are available from the XXX Institutional Data Access / Ethics Committee (contact via XXX) for researchers who meet the criteria for access to confidential data.*<br><br>*The data underlying the results presented in the study are available from (include the name of the third party*	All relevant data are within the manuscript and its Supporting Information file 2.

Powered by Editorial Manager® and ProduXion Manager® from Aries Systems Corporation

and contact information or URL).<br>• This text is appropriate if the data are owned by a third party and authors do not have permission to share the data.<br><br>* typeset	
Additional data availability information:	

Main document MS

Click here to access/download;Manuscript;Fertility_antqueens_250804_LJ.d

Fertility correlates with queen size and sperm quality in an ant

Authors: Süß, Lena-Marie[1]; Oettler, Jan[1]; Jaimes-Nino, Luisa-Maria[1,2]

Affiliation: [1]Zoologie/Evolutionsbiologie, Universität Regensburg, 93053 Regensburg, Germany

[2] Behavioral Ecology and Social Evolution Group, Institute of Organismic and Molecular Evolution, Johannes Gutenberg University, 55128 Mainz, Germany.

ORCIDs:

Luisa Maria Jaimes-Nino: 0000-0003-1186-1837

Jan Oettler: 0000-0002-8539-6029

Corresponding author: Jaimes-Nino, LM in jaimes.luisa@outlook.com

Number of words (without references): 4300

Number of Figures: 4

Supplementary files: 3 (Supplementary File 1 – Figures, Supplementary File 2 – Data, Supplementary File 3 – R Script)

Abstract

Fitness can vary between individuals of the same population for reasons related to nutrient acquisition during development, but also to the quality of the mating partners. We focus on ant queens of *Cardiocondyla obscurior* to investigate the effects of female and male morphology and sperm characteristics on productivity. We monitored queens for 12 weeks after mating as a proxy for lifetime productivity, and found that larger queens are more productive, as commonly found in other insects. Sperm viability, but not sperm length, was positively correlated with female-biased sex ratios, pointing to a better insemination rate. The high correlation between sperm viability, male body size and mandible size suggests strong selection for larger and stronger males. However, male competition for access to unmated queens in the maternal colony may result in a trade-off between male size and developmental time in this species.

Keywords: caste ratio, fecundity, sex ratio, social insects, sperm traits

Introduction

Females generally invest a substantial part of their resources in the embryonic development of their offspring, while the male's contribution is typically confined to the quantity and quality of transferred sperm and seminal fluids. In relation to costly female resources in reproduction, a well-documented factor influencing maternal fertility in insects, i.e. demonstrated fecundity, is female body size (1,2). Adult body size is determined genetically and modified by the quantity or quality of provisioning during immature stages, or variation in developmental time (3,4). In holometabolous insects, adult body size is largely determined during the larval stage highlighting the role of the developmental environment in shaping reproductive outcomes (5). In terms of male fitness traits, body size exhibits a stronger condition-dependency on nutritional limitations during certain life stages than ejaculate traits (6), suggesting that the sperm-related traits are strongly constrained and selected to maintain reproductive function. Generally, nutrient restriction greatly reduces the quantity of seminal fluid and sperm, while the quality of sperm, specifically viability and morphology, is less consistently affected. These critical variables appear to be strongly canalized during development, enabling even low-condition males to achieve fertilization (6). Alternatively, the costs and resource investment for ejaculate traits might be lower compared to those for body size, thereby reducing their dependence on environmental conditions.

In insects where copulation involves a single mating event, the lifetime supply of sperm is retained and maintained in a specialized organ called the spermatheca (7,8). Spermathecal gland proteins and seminal fluids sustain a high-quality pool of viable spermatozoa, by reducing oxidative stress and cellular senescence (9–11). Further, spermiophagy during long-term sperm storage eliminates dead sperm cells which are a potential source of toxic molecules, and enables a more efficient fertilization of eggs (9,12–14). In the social Hymenoptera, male body size is apparently related to greater sperm transfer and mating success in *Pogonomyrmex occidentalis* (15,16), but negatively with sperm counts in *Atta colombica* (17). This suggests different selection mechanisms operating in species where females mate with multiple males.

The ant species *Cardiocondyla obscurior* is characterized by polygynous colonies and intranidal mating of queens with wingless males that have long, sickle-shaped mandibles for killing rivals (18–22). The wingless male morph maintains lifelong spermatogenesis, allowing them to replenishing their sperm supply and mate with multiple females during their relatively long lives, whereas queens typically mate with a single male (19,20,23). Queen fertility, based on egg productivity, is positively correlated with lifespan (24,25), and longer-lived queens also produce more sexuals (24).

However, variation in lifespan and fertility among queens is high (24,25), and the causes of such variation remain unknown. *C. obscurior* brood is reared in loose piles, with

eggs and pupae separated from larvae to prevent cannibalism. Additionally, larvae should experience the same environment and worker care in the nest. Given this presumed lack of variation in extrinsic cues, we sought to investigate other factors influencing differences in offspring number, sex and caste allocation, and ultimately female fecundity. We hypothesized that maternal condition has a strong effect on the fertility of *Cardiocondyla obscurior* ant queens while paternal condition does not. However, our findings indicate that queen fertility is correlated to paternal body size of both females and males. Larger queens were also more fertile and produced a more female-biased offspring ratio when mated to larger males with more viable sperm.

Methods

Model organism

The *Cardiocondyla obscurior* (Formicidae: Myrmicinae) tramp ant forms small colonies ranging from a few workers up to 150 (median = 28.5, SD = 31.4, n =62) in their natural habitat (24). The species originates from Southeast Asia and is widely distributed throughout the tropics and subtropics. It is notable for its male diphenism, with a wingless ergatoid (from Greek ergat,-'worker' and oid, -'less') male equipped with long, sickle-shaped mandibles which they employ in lethal fights with rival males to monopolize reproduction, and winged males that are occasionally found (21,26,27). In contrast to wingless males, the winged male morph completes spermatogenesis upon adult emergence and produces shorter, less variable sperm, though their sperm viability does not differ from that of wingless males (28). The ants used here were collected in 2011 in Japan (29,30). Since then, the ants were propagated in the laboratory, fed ad libitum with honey, cockroaches and flies three times per week, and kept in a climate-controlled room on a 12 h dark 22°C/12 h light 26°C cycle, at 75-80% humidity.

Female and male effects on fertility

Queens mate with a single male and have control over caste and sex allocation (31), making it possible to monitor lifetime investment of single queens (24). Individual colonies (n=57) were set up with a dark-colored queen pupa and an adult wingless male of unknown age collected from a different stock colony. Male age is not known to influence sperm quality or offspring production in this species (32,33). Four freshly eclosed "callow" workers, and four L3 last instar worker larvae were added to start the colony, collected from the male’s colony to avoid any worker behavior that could be elicited by an alien odor. Once the queen started egg-laying the larvae were removed, and the number of workers was increased to 15 and subsequently

standardized three times per week. Eggs, larvae, workers, and pupae were counted weekly, and pupated individuals were removed three times per week. After 12 weeks of the start of egg laying (adult age range 9 – 23 days) the queen was frozen at -20°C, and the colony was further monitored as described above until all larvae eclosed. Of 57 F1 queens, 42 (73.7%) successfully mated, however, 3 queens died before the end of the observation period of 12 weeks and were not included in the correlation analyses.

Sperm viability and length

Males from each successful pairing (n=39) were dissected on a microscope slide in a drop of Beadle solution (128.3 mM NaCl, 4.7 mM KCl, 2.3 mM $CaCl_2$) and the reproductive tissue was transferred to 10 µl fresh Beadle solution. The sperm cells were then released by opening the seminal vesicles and accessory glands (Supplementary Figure 1A-C). Sperm viability was assessed with a LIVE/DEAD sperm viability kit (Molecular Probes, Eugene, Oregon, USA). For staining, 5 µl of SYBR-14 working solution (SYBR stock solution diluted 1:50 in Beadle solution) was added and mixed with the sperm sample. After a 10-minute incubation in a humidity chamber under exclusion of light, 2 µl of propidium iodide was added, mixed in carefully, and incubated in the dark for 7 minutes. Prior to microscopy, a coverslip was placed on the sample droplet. The sperm samples were then examined by fluorescence microscopy (Axiophot, Zeiss, Germany), taking images of 5 randomly selected areas of the sample (20x magnification). For the analysis, the images were blinded and all live (green) and dead (red) sperm cells of the 5 images were counted, and a proportion was calculated for each male. After determination of sperm viability, measurements for sperm length were assessed by photographing the slides in transmitted light at 40x magnification in 5 randomly selected areas. The length of 10 random sperm cells per picture was measured blindly for each sample using ImageJ (34), tracing a freehand line from head to tail end. For each male, the median sperm length and the standard deviation were calculated across the 5 images.

Female and male morphometrics

Heads and thoraxes of F0 queens and males were measured using a VHX-5000 digital microscope (20x to 200x objective; Keyence) and analyzed using ImageJ. The morphometric images were blinded before evaluation. For queens, head width (QHW) and length (QHL), thorax width (QTW) and length (QTL), and spine distance (QSpD) were measured. For wingless males, head width (MHW) and length (MHL), thorax width (MTW) and length (MTL),

left and right mandible length (MDL and MDR), petiole width, and antennal segment number were determined. The head was measured in frontal view, length results from the anteriormost point of the clypeus to the posteriormost point of the head, and width represents the distance above the eyes. The thorax was measured in dorsal view, length measured from the mesoscutellum to the end of the mesosoma, width in the queens equals the widest point of the mesoscutellum in front of the wings, and in males at the widest point of the thorax. The distance between the spines was also measured in dorsal view.

Statistical analyses of female and male effects

The predictor variables used to correlate to the queen's fertility were selected based on their high coefficient of variance and low correlation with other variables (see results section). The correlation matrix for the morphometric data for both, queens and males, was calculated using Pearson Product Moment correlation coefficient *cor.test* (stats R package) for normally distributed data and Kendall rank correlation coefficient Kendall's τ *cor* (stats R package v. 4.4.1) for non-normally distributed data. Significant p-values were obtained using *cor.mtest* [corrplot R package v. 0.95, (35)] and *ggcorrplot* [ggcorrplot R package v. 0.1.4, (36)] for visualization purposes.

Fitted regression models were performed with QTL, median sperm length, and sperm viability, as predictor variables. We fit models with gaussian distribution using glmmTMB (R package v. 1.1.10, (37), to analyze the effect on queen fertility, that is, for the total sum eggs produced, sum of larvae produced, worker pupae, and total pupae counted as

~ QTL + median_µm + percentage_live

When investigating the effect on sexual offspring, the time of sexual production (queen or male pupae production) was included in the model because some queens started producing sexuals at the end of the tracking period of 12 weeks, leaving a shorter time window of data collection. We decided to include the time of sexual production (in weeks) as a continuous covariate when the difference between model AIC was >2. We modelled the production of queen pupae, ergatoid pupae, winged male pupae, and caste ratio (queens/(queens+workers)) as

~ QTL + median_µm + percentage_live + time_sexual_pupae_production

with a gaussian distribution using glmmTMB unless specified otherwise. The model to fit the production of winged male pupae used a negative binomial generalized linear model (nbinom2), and the predictor variable "time of sexual production" was dropped because the improvement in AIC was <2.

Further, we investigated the effect on sex ratio specifying the response as *cbind*(queen pupae, male pupae) and a binomial distribution family (glmmTMB) as

sex ratio ~ QTL + median_μm + percentage_live

and dropping the time of sexual production as covariate because the improvement in AIC was <2. All models were checked for correct distribution (KS test), dispersion and outliers using the function *simulateResiduals* [DHARMA package version 0.4.6, (38)]. Lastly, we tested the effect of maternal size on the male type (winged and wingless males vs only wingless males) and on the sex type (queens and males or queens and wingless males vs only queens) of sexual offspring they produced using a Kruskal-Wallis test (stats R package), and post-hoc pairwise comparison using Dunn's test corrected for a false discovery rate [dunn.test R package v.1.3.6., (39)]. All tests and graphs were performed using R [v. 4.4.3 (40)].

Results

Higher trait correlation in queens than in males

The body measurements of the queens were all highly significantly correlated to each other (n = 42, τ, $p < 0.001$; Figure 1A), therefore QTL was used as the representative variable for queen body size in all the regression models (coefficient of variance, $CV_{QHW} = 1.90$, $CV_{QHL} = 2.14$, $CV_{QTW} = 3.00$, $CV_{QTL} = 2.65$, $CV_{QSpD} = 5.41$). The male morphometric data were not as strongly correlated as those of the queens (Figure 1B) and showed greater overall variation (n = 42, $CV_{MHW} = 3.69$, $CV_{MTW} = 4.41$, $CV_{MTL} = 4.47$, $CV_{MDL} = 4.45$, $CV_{MDR} = 3.88$).

Figure 1. Morphometric correlation matrix. Computed Kendall rank correlation coefficients of A) queens (n = 42) for thorax width (QTW), thorax length (QTL), head length (QHL), head width (QHW), and spine distance (QSpD). All the performed morphometric correlations were highly significant (τ, $p < 0.001$), and wingless (ergatoid) males (n=42) for thorax width (MTW), thorax length (MTL), head length (MHL), head width (MHW), mandible length left (MDL) and right (MDR), sperm viability (Viab), as well as the median sperm length (Median_L), standard deviation (SD_L) and coefficient of variance of sperm length (CV_L). The correlation coefficient ranges from a strong negative correlation (r = 1.0, blue) to a strong positive correlation (r = 1.0, red). Only significant correlations ($p < 0.05$) are shown.

Larger queens are more productive

Queen thorax length (QTL) was positively correlated to the number of eggs (glmmTMB, z = 1.97, p = 0.04), larvae (glmmTMB, z = 2.90, $p < 0.01$) and the total number of pupae produced

(glmmTMB, $z = 2.54$, $p = 0.011$, Figure 2A, B). An increase of 1 um of queen thorax corresponded to 3.7 (SE±1.3) more larvae and 3.3 (SE±1.3) more pupae been observed in the span of 12 weeks since egg laying started. The production of worker pupae was also positively correlated with queen size (glmmTMB $z = 2.74$, $p < 0.01$; Figure 2C). Similarly, an increase of 1 um of queen thorax corresponded to 3.2 (SE±1.2) more worker pupae being produced.

Figure 2. Correlation of queen body size with fertility (n=39). A statistically significant positive correlation was found between queen's thorax length (QTL) and the total A) pupae (light blue), egg (black), B) larvae, and C) worker pupae production. A positive correlation was found between QTL and D) the production of winged male pupae (green), with no significant effect on wingless male pupae ('ergatoid', dark blue) production. No difference in queen production (Suplementary Figure 2) translates in E) a lower caste ratio (queens/queens+workers), and F) a lower sex ratio (queens/queens+males) for larger queens.

The onset of sexual production had a significant effect on the number of wingless males (glmmTMB, $z = -2.75$, $p < 0.01$, Figure 3A), queen pupae produced (glmmTMB, $z = -3.89$, $p < 0.001$, Figure 3B), and the caste ratio produced (glmmTMB, $z = -2.94$, $p < 0.01$, Figure 3C). The earlier queens started sexual production, the more wingless male and queen pupae were produced in the span of the experiment. Therefore, we included the time of sexual production as covariate when modelling the total pupae production separately by sex and caste. We found that larger queens (higher QTL) produce more winged male pupae (glmmTMB, $z = 2.13$, $p = 0.033$; Figure 2D), and a similar number of wingless pupae (glmmTMB $z = 0.84$, $p = 0.40$; Figure 2D). The number of queens produced did not correlate with queen size (glmmTMB, $z = -1.08$, $p = 0.28$; Supplementary Figure 2). Therefore, larger queens have a stronger worker-biased caste ratio (queens/queens+workers, glmmTMB, $z = -2.41$, $p = 0.02$, Figure 2E), with an increase in 1 um corresponding to a decrease of 0.1% (SE±0.04%) of caste ratio during the 12 weeks of censoring. Similarly, queen size (QTL) was negatively correlated with sex ratio (queens/queens+males) (glmmTMB, $z = -2.37$, $p = 0.02$, Figure 2F). The odds of a pupae being a queen (vs. male) decreases by about 2% with an increase of 1 um of queen thorax.

Figure 3. Onset of sexual production. Offspring production was monitored 12 weeks after egg laying started, but queens varied in the onset of sexual production. The earlier a queen

started producing queen pupae, the more A) wingless male pupae but not winged male pupae were produced. B) The timing of sexual production was correlated with B) queen pupae production and, C) the caste ratio (queens/queens+workers).

QTL was significantly greater in queens producing both ergatoid and winged male pupae (median$_{(Q+E+WM)}$ =579.47 mm, range = 34.85mm) compared to queens producing only ergatoid pupae (median$_{(Q+E)}$ = 560.35mm, range = 49.92mm; Kruskal-Wallis test χ_2^2 = 0.037, p = 0.037; post-hoc Dunn's test z = 2.55, p = 0.033; Supplementary Figure 3). Queens producing only female sexuals were less fertile than queens that also produced male sexuals. Queens produced a median of 152 eggs when they only produced female offspring, compared to 209.5 eggs when producing queens and wingless males together (Kruskal-Wallis test χ_2^2 = 10.29, p = 0.006; post-hoc Dunn's test z (Q vs. Q+E) = 2.18, p(Q vs. Q+E) = 0.043 and z(Q vs Q+E+WM) = 3.21, p(Q vs Q+E+WM) = 0.004, Supplementary Figure 4).

Sperm viability leads to female-biased caste ratios

A total of 1149 spermatozoa from 39 wingless males were measured and checked for viability. Sperm viability positively correlated with sex ratio (queens/queens+males; binomial glmmTMB OR = 12.5, 95% CI [2.62, 59.4], z = 3.17, p < 0.01, Figure 4A). Similar results were obtained when considering all the female offspring (females/females + males; binomial glmmTMB OR = 4.08, 95% CI [1.14, 14.7], z = 2.15, p = 0.031). There was a tendency in males with a higher sperm viability to sire less wingless males (glmmTMB, z= -1.91, p = 0.056, Figure 4B).

The sperm quality did not affect other fertility traits as the production of eggs (glmmTMB, median sperm length: z = - 0.91, p = 0.364; viability: z = - 0.99, p = 0.321), winged male pupae (sperm length: z = - 1.09, p = 0.274; viability: z = 0.09, p = 0.932), wingless male pupae (sperm length: z = 0.83, p = 0.404), queen pupae (sperm length: z = - 0.76, p = 0.447; viability: z = 1.06, p = 0.288) or worker pupae (sperm length z = - 1.59, p = 0.113; viability: z = 0.03, p = 0.980). Median sperm length did not affect sex ratio (glmmTMB binomial z = - 0.50, p = 0.620), and the caste ratio was not affected by sperm quality (sperm length, z = 0.25, p = 0.806; viability: z = 1.11, p = 0.27).

Figure 4. Influence of male sperm viability on offspring production (n=39). A) Higher sperm viability correlated significantly with a stronger female-biased sex ratio

(queens/queens+males). B) A tendency was found between the sperm viability and the negative production of wingless males.

Discussion

As observed in several insect species (2), maternal body size is positively correlated with fertility, i.e. the production of eggs, larvae, and total pupae. Larger queens produced more worker pupae and winged male pupae, but similar numbers of queen pupae. Hence, at a colony level larger queens invest more into the workforce, while all queens invest equally to the production of female sexual pupae. This is true for the first twelve weeks since egg laying started, which is a good predictor for lifetime egg production (41). In this species lifetime egg production and sexual offspring are positively correlated (24,25), and production of sexuals reaches a maximum at around 30 weeks (24). Consequently, it is likely that larger queens will end up producing more sexuals once the ergonomic phase of the colony is surpassed, i.e. the phase of colony growth with main investment in the workforce.

While larger queens in polygynous colonies might be responsible for producing most of the workforce, they are the only ones producing winged males. It possible that the production of winged males is more energetically costly compared to the production of the wingless morph. Most of the males produced in single-queen colonies of *C. obscurior* are wingless [10% winged vs 90% wingless, (24)]. Winged male development from egg to adult is one third longer than wingless males [winged 30 days vs 20 days wingless, (42)], and similarly long as queens. Further, the male morphs differ in body size, reproductive tactics, lifespan, sperm length, and accessory gland proteins (26,28,42–44). Both morphs mate intranidal with closely related females (26), but winged males, considered the dispersal morph, leave the nest later in search of unrelated females (45). Winged males live a few days to weeks while wingless males live up to three months (23,46). Compared to queens, males in *C. obscurior* exhibit greater reproductive variance as some males will sire a high number of offspring, while many males die in combat and sire none. Theory predicts that parents in good condition should preferentially produce the sex with the higher reproductive variance, whereas lower condition individuals should produce the sex with the lower reproductive variance (47,48). Larger queens may therefore have more resources for high-risk investment into male production than smaller queens.

In contrast, sperm length in wingless males did not correlate with total fertility, measured by the number of eggs. However, sperm viability was positively associated with queen-biased sex ratios. In social insects, strong selection for high-quality ejaculates is expected due to the prolonged storage of sperm in the spermatheca (8) and the significant

immunological costs to females associated with sperm storage (7,9). Hymenoptera are haplodiploid, with males developing from unfertilized haploid oocytes. Therefore, ejaculates with more viable sperm will achieve higher insemination rates. Consistent with this, we found that females mated to males with low sperm viability tended to produce more male offspring. Sperm viability is predicted to be higher in species with more intense sperm competition. Evidence in several insect taxa support this hypothesis, as monandrous species exhibit a lower proportion of live sperm (49), and sperm viability influences paternity in polyandrous species (50). Sperm viability might be under strong selection in polyandrous species, where male post-ejaculatory competition is strong. In our model organism, females mate only once and male competition occurs predominantly before mating, suggesting a relaxed post-ejaculatory selection on sperm viability.

The wingless male morph is an evolutionary novelty in the genus *Cardiocondyla* and the ancestral winged male morph has been lost convergently (21). Unlike males of most ant species, wingless males are relatively long-lived, and capable to mate with multiple females (23). Their spermatogenesis continues during their adult life, allowing for frequent replenishment of the sperm supply (19,28). Paternal trait correlations showed that larger wingless males had more viable sperm. Further, head and thorax size positively correlated with mandible length, used to immobilize rivals during combat. Such fights can last for over a day, ending when the stronger male chemically marks the opponent with hindgut secretions, to which workers react and eliminate the defeated rival (18). However, males with longer mandibles and larger body size may be outcompeted if their developmental time exceeds that of their competitors. Smaller males with faster developmental times may outcompete slower-developing rivals by targeting pupae and newly eclosed males (26). For instance, in a parasitic wasp, early emergence confers a fighting advantage among males of equal size (51). Since smaller body size correlates with reduced sperm viability, male size and sperm quality may be under trade-off due to strong sexual selection. Morphological traits exhibited a higher variation in males compared to queens, suggesting variation in the optimal male morphology.

In summary, during the ergonomic phase of colony growth, colonies with large queens can grow faster due to increased investment in worker production. Larger queens are also more likely to produce a greater number of sexuals, producing winged males that disperse and more queen pupae in the later phases of colony development. The size of wingless males is constrained by opposing selective pressures. While larger males achieve higher insemination rates and sire more female offspring, their extended developmental time makes them more vulnerable to rivals, assuming growth rate remains constant.

Supporting materials

Supplementary File 1 – Supplementary Figures

Supplementary File 2 – Excel file with raw data and summarized data (XLSX).

Supplementary File 3 – R Script to replicate the results of the study.

Acknowledgments

We would like to thank Thomas Anthony Keaney for helpful comments. We would like to thank Leonie Riemer for the morphological measurements. We also would like to thank Federico Olivera-Rodriguez, Benjamin Dofka, Melanie Schlossberger, Leonard Mecka, and Maike Fallböhmer for helping maintain the colonies. Finally, we want to thank the DFG Research Unit FOR2281.

Author contributions

LMS: Methodology, Data curation, Formal analysis, Writing – original draft, Writing – review & editing. JO: Conceptualization, Funding acquisition, Writing – original draft, Writing – review & editing. LMJN: Conceptualization, Formal analysis, Funding acquisition, Project administration, Supervision Validation, Writing – original draft, Writing – review & editing.

Funding

This research was supported by a grant from the FAS-G (Finanzielles Anreizsystem zur Förderung der Gleichstellung) of the University of Regensburg to LMJN., and grants of the Deutsche Forschungsgemeinschaft to JO and LMJN (OE549/2-2,3, JA3614/1-1). No funding sources were involved in study design, data collection and interpretation, or the decision to submit the work for publication.

Competing interest

The authors declare no competing interests.

Data accessibility

All data sets to reproduce the figures and results are available as supplementary material (Supplementary File 2 and 3).

References

1. Blanckenhorn WU. The Evolution of Body Size: What Keeps Organisms Small? The Quarterly Review of Biology. 2000;75(4):385–407.

2. Honěk A. Intraspecific Variation in Body Size and Fecundity in Insects: A General Relationship. Oikos. 1993;66(3):483–92.

3. Chown SL, Gaston KJ. Body size variation in insects: A macroecological perspective. Biological Reviews. 2010;85:139–69.

4. Teder T, Kaasik A, Taits K, Tammaru T. Why do males emerge before females? Sexual size dimorphism drives sexual bimaturism in insects. Biological Reviews. 2021;96(6):2461–75.

5. Hanna L, Lamouret T, Poças GM, Mirth CK, Moczek AP, Nijhout FH, et al. Evaluating old truths: Final adult size in holometabolous insects is set by the end of larval development. Journal of Experimental Zoology Part B: Molecular and Developmental Evolution. 2023;340(3):270–6.

6. Macartney EL, Crean AJ, Nakagawa S, Bonduriansky R. Effects of nutrient limitation on sperm and seminal fluid: a systematic review and meta- analysis. Biological Reviews. 2019 Oct;94(5):1722–39.

7. Baer B, Boomsma JJ. Mating biology of the leaf-cutting ants *Atta colombica* and *A. cephalotes*. J Morphol. 2006 Oct;267(10):1165–71.

8. Boomsma JJ, Baer B, Heinze J. The Evolution of Male Traits in Social Insects. Annual Review of Entomology. 2005;50(1):395–420.

9. Degueldre F, Aron S. Long- term sperm storage in eusocial Hymenoptera. Biological Reviews. 2023 Apr;98(2):567–83.

10. den Boer SPA, Sturup M, Boomsma JJ, Baer B. The ejaculatory biology of leafcutter ants. Journal of Insect Physiology. 2015 Mar;74:56–62.

11. den Boer SPA, Boomsma JJ, Baer B. Honey bee males and queens use glandular secretions to enhance sperm viability before and after storage. Journal of Insect Physiology. 2009 June 1;55(6):538–43.

12. den Boer SPA, Baer B, Dreier S, Aron S, Nash DR, Boomsma JJ. Prudent Sperm Use by Leaf-Cutter Ant Queens. Proceedings: Biological Sciences. 2009;276(1675):3945–53.

13. Paynter E, Millar AH, Welch M, Baer-Imhoof B, Cao D, Baer B. Insights into the molecular basis of long-term storage and survival of sperm in the honeybee (*Apis mellifera*). Sci Rep. 2017 Jan 16;7(1):40236.

14. Poland V, Eubel H, King M, Solheim C, Harvey Millar A, Baer B. Stored sperm differs from ejaculated sperm by proteome alterations associated with energy

metabolism in the honeybee *Apis mellifera*. Molecular Ecology. 2011 June;20(12):2643–54.

15. Abell AJ, Cole BJ, Reyes R, Wiernasz DC. Sexual selection on body size and shape in the Western Harvester Ant, *Pogonomyrmex occidentalis* Cresson. Evolution. 1999;53(2):535–45.

16. Wiernasz DC, Sater AK, Abell AJ, Cole BJ. Male Size, Sperm Transfer, and Colony Fitness in the Western Harvester Ant, *Pogonomyrmex occidentals*. Evolution. 2001;55(2):324–9.

17. Fjerdingstad EJ, Boomsma JJ. Variation in size and sperm content of sexuals in the leafcutter ant Atta colombica. Insectes soc. 1997 Aug 1;44(3):209–18.

18. Yamauchi K, Kawase N. Pheromonal manipulation of workers by a fighting male to kill his rival males in the ant *Cardiocondyla wroughtonii*. Naturwissenschaften. 1992;79(6):274–6.

19. Heinze J, Hölldobler B. Fighting for a harem of queens: physiology of reproduction in *Cardiocondyla* male ants. Proceedings of the National Academy of Sciences of the United States of America. 1993;90(September):8412–4.

20. Heinze J, Hölldobler B, Yamauchi K. Male competition in *Cardiocondyla* ants. Behav Ecol Sociobiol. 1998 Apr 1;42(4):239–46.

21. Oettler J, Suefuji M, Heinze J. The evolution of alternative reproductive tactics in male *Cardiocondyla* ants. Evolution. 2010;64(11):3310–7.

22. Schmidt CV, Schrempf A, Trindl A, Heinze J. Microsatellite markers for the tramp ant, *Cardiocondyla obscurior* (Formicidae: Myrmicinae). Journal of Genetics. 2016;95(December):1–4.

23. Metzler S, Heinze J, Schrempf A. Mating and longevity in ant males. Ecol Evol. 2016 Dec;6(24):8903–6.

24. Jaimes-Nino LM, Heinze J, Oettler J. Late-life fitness gains and reproductive death in *Cardiocondyla obscurior* ants. eLife. 2022;11:1–17.

25. Kramer BH, Schrempf A, Scheuerlein A, Heinze J. Ant colonies do not trade-off reproduction against maintenance. PLoS ONE. 2015;10(9):1–13.

26. Heinze J. Life-history evolution in ants: the case of *Cardiocondyla*. Proceedings of the Royal Society B: Biological Sciences. 2017;284(1850):20161406.

27. Heinze J, Trindl A, Seifert B, Yamauchi K. Evolution of male morphology in the ant genus *Cardiocondyla*. Molecular Phylogenetics and Evolution. 2005;37(1):278–88.

28. Schrempf A, Moser A, Delabie J, Heinze J. Sperm traits differ between winged and wingless males of the ant *Cardiocondyla obscurior*. Integrative Zoology. 2016;11(6):427–32.

29. Errbii M, Keilwagen J, Hoff KJ, Steffen R, Altmüller J, Oettler J, et al. Transposable elements and introgression introduce genetic variation in the invasive ant *Cardiocondyla obscurior* . Molecular Ecology. 2021;(July):1–18.

30. Schrader L, Kim JW, Ence D, Zimin A, Klein A, Wyschetzki K, et al. Transposable element islands facilitate adaptation to novel environments in an invasive species. Nature Communications. 2014;5:1–10.

31. Schultner E, Wallner T, Dofka B, Brülhart J, Heinze J, Freitak D, et al. Queens control caste allocation in the ant *Cardiocondyla obscurior*. Proceedings of the Royal Society B: Biological Sciences. 2023 Feb 8;290(1992):20221784.

32. Heinze J, Hanoeffner M, Delabie JHC, Schrempf A. Methuselah's daughters Paternal age has little effect on offspring number and quality in *Cardiocondyla* ants. Ecology and Evolution. 2018;00:1–7.

33. Metzler S, Schrempf A, Heinze J. Individual- and ejaculate-specific sperm traits in ant males. Journal of Insect Physiology. 2018 May 1;107:284–90.

34. Schneider CA, Rasband WS, Eliceiri KW. NIH Image to ImageJ: 25 years of image analysis. Nat Methods. 2012 July;9(7):671–5.

35. Taiyun W, Vilian S. R package 'corrplot': Visualization of a Correlation [Internet]. 2025 [cited 2025 Mar 31]. Available from: https://github.com/taiyun/corrplot

36. Kassambara A. GitHub - kassambara/ggcorrplot: Visualization of a correlation matrix using ggplot2 [Internet]. 2022 [cited 2025 Mar 31]. Available from: https://github.com/kassambara/ggcorrplot

37. Brooks ME, Kristensen K, van Benthem KJ, Magnusson A, Berg CW, Nielsen A, et al. glmmTMB balances speed and flexibility among packages for zero-inflated generalized linear mixed modeling. The R journal. 2017 Dec;9(2):378–400.

38. Hartig F. DHARMa : Residual Diagnostics for Hierarchical (Multi-Level / Mixed) Regression Models [Internet]. R package; 2020. Available from: https://cran.r-project.org/package=DHARMa

39. Dinno A. dunn.test: Dunn's Test of Multiple Comparisons Using Rank Sums [Internet]. 2014 [cited 2025 Mar 31]. p. 1.3.6. Available from: https://CRAN.R-project.org/package=dunn.test

40. R Core Team. R: A language and environment for statistical computing [Internet]. Vienna, Austria; 2024 [cited 2024 Oct 31]. Available from: https://www.r-project.org/

41. Jaimes- Nino LM, Oettler J. No correlation between mother and daughter life-history traits in *Cardiocondyla obscurior*. Insectes Sociaux. 2025;72(1):1–9.

42. Schrempf A, Heinze J. Proximate mechanisms of male morph determination in the ant *Cardiocondyla obscurior*. Evolution and Development. 2006;8(3):266–72.

43. Fuessl M, Santos CG, Hartfelder K, Schrempf A, Heinze J. Accessory gland proteins of males in the male-diphenic ant *Cardiocondyla obscurior*. Physiological Entomology. 2018 Dec;43(4):276–84.

44. Kinomura K, Yamauchi K. Fighting and mating behaviors of dimorphic males in the ant - *Cardiocondyla wroughtoni*. Journal of Ethology. 1987;5(1):75–81.

45. Cremer S, Schrempf A, Heinze J. Competition and opportunity shape the reproductive tactics of males in the ant Cardiocondyla obscurior. PLoS ONE. 2011;6(3):14–6.

46. Heinze J. The male has done his work — the male may go. Current Opinion in Insect Science. 2016;16:22–7.

47. Carl V, Haig D, Nowak MA. The Trivers–Willard hypothesis: sex ratio or investment? Proceedings of the Royal Society B: Biological Sciences [Internet]. 2016 [cited 2024 Aug 1];283. Available from: https://royalsocietypublishing.org/doi/epdf/10.1098/rspb.2016.0126

48. Trivers RL, Willard DE. Natural Selection of Parental Ability to Vary the Sex Ratio of Offspring. Science. 1973 Jan 5;179(4068):90–2.

49. Hunter FM, Birkhead TR. Sperm Viability and Sperm Competition in Insects. Current Biology. 2002 Jan;12(2):121–3.

50. García-González F, Simmons LW. Sperm Viability Matters in Insect Sperm Competition. Current Biology. 2005 Feb;15(3):271–5.

51. Innocent TM, Savage J, West SA, Reece SE. Lethal combat and sex ratio evolution in a parasitoid wasp. Behavioral Ecology. 2007 July 1;18(4):709–15.

Figure 1.

Click here to access/download;Figure;Fig1_correlations.pdf

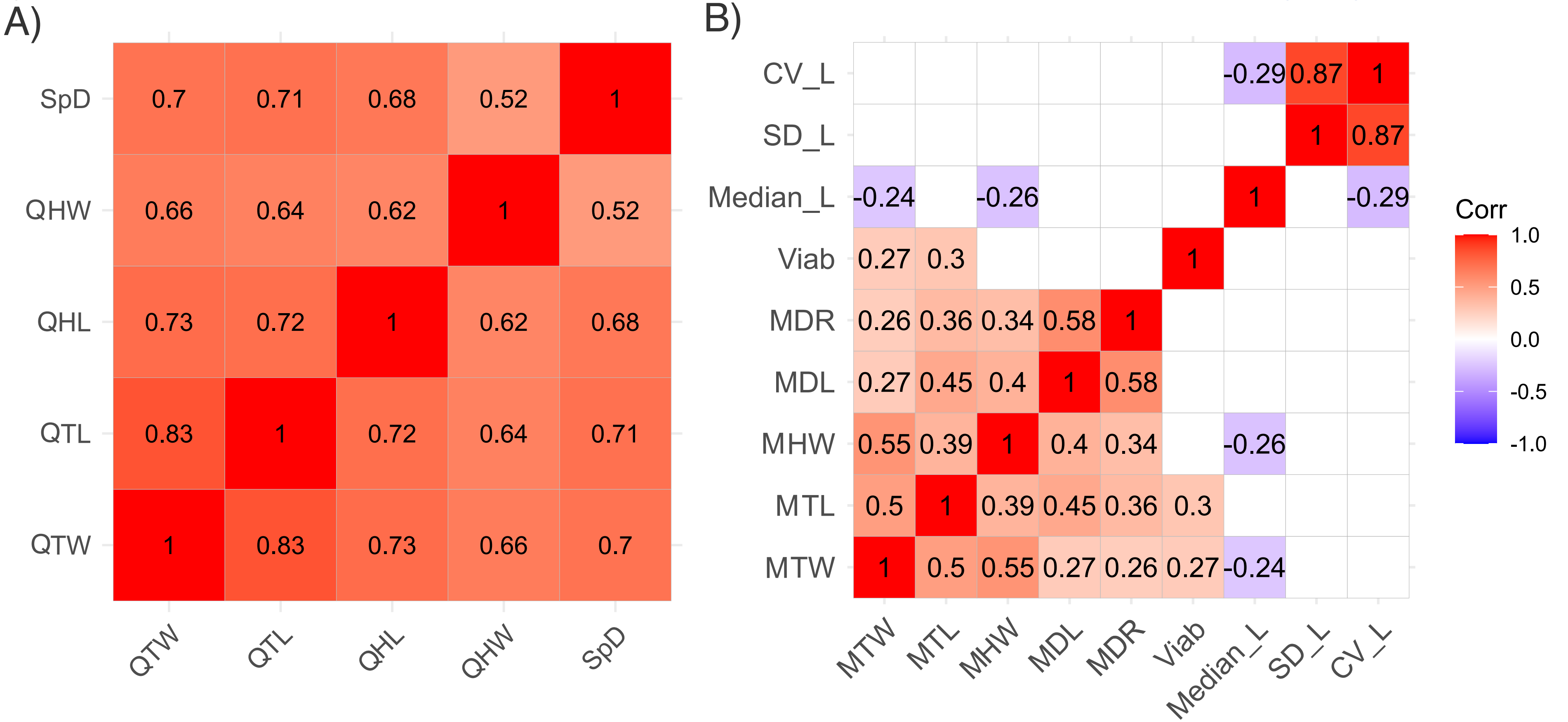

Figure 2

Click here to access/download;Figure;Fig2_QTL_offspring.pdf

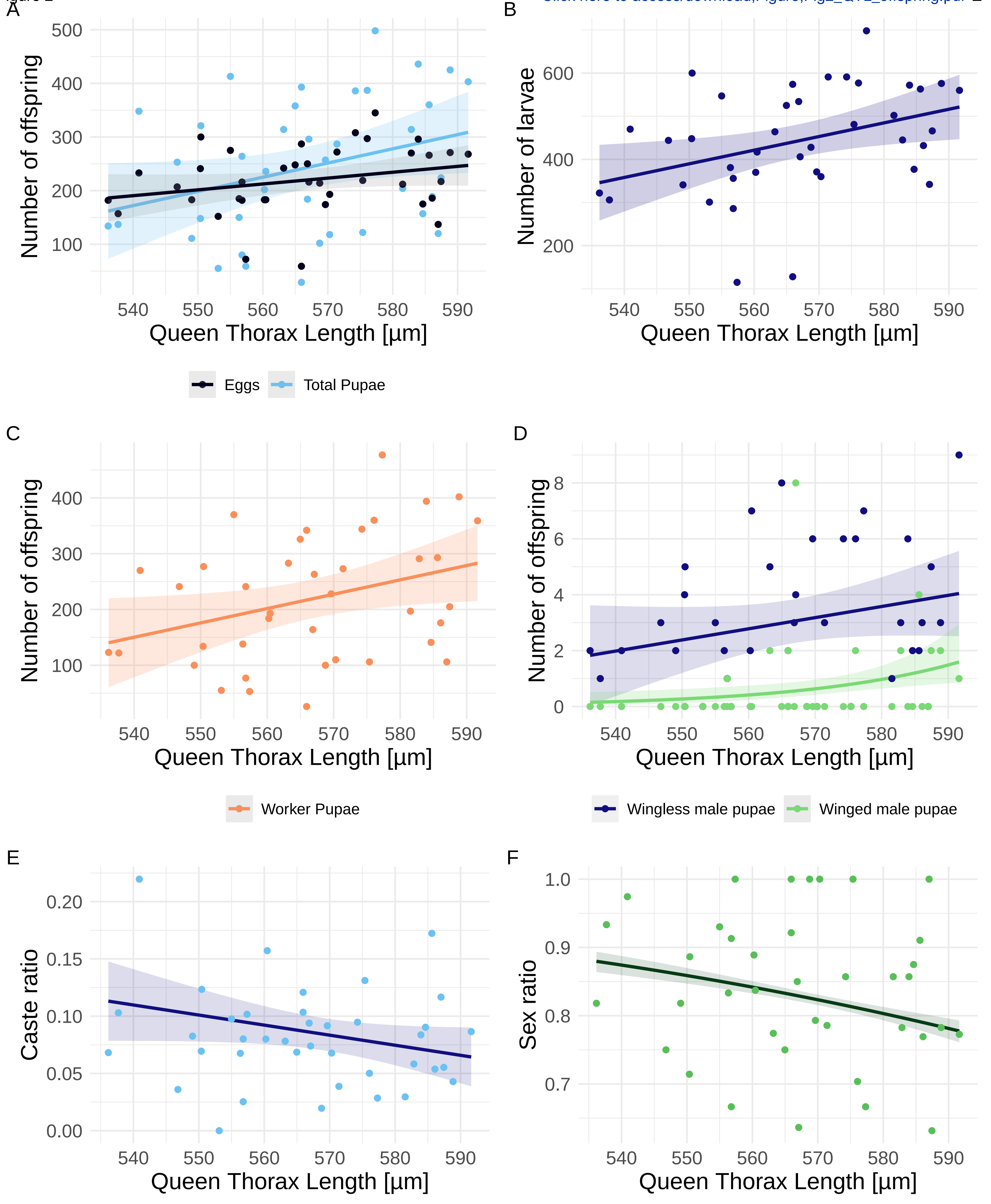

Figure 3

Click here to access/download;Figure;Fig3_onset_sexual_prod.pdf

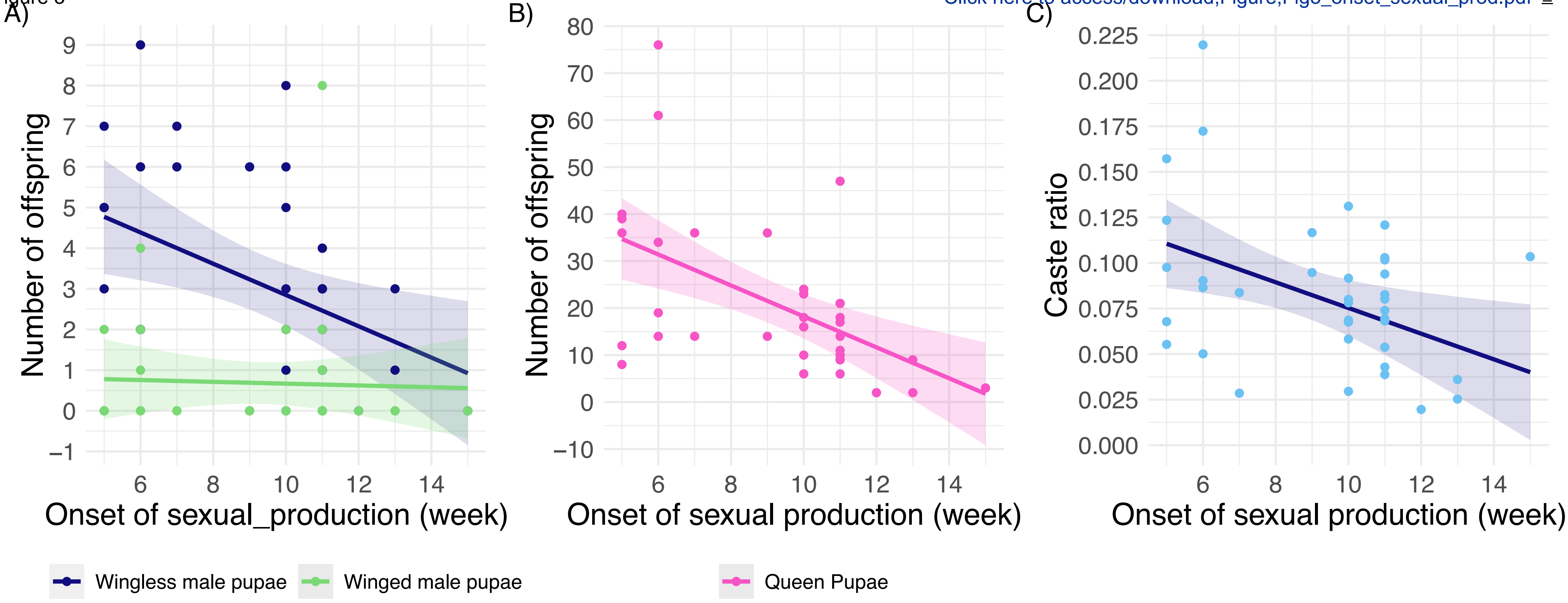

Figure 4

Click here to access/download;Figure;Fig4_Spermviab_sexRatio.pdf

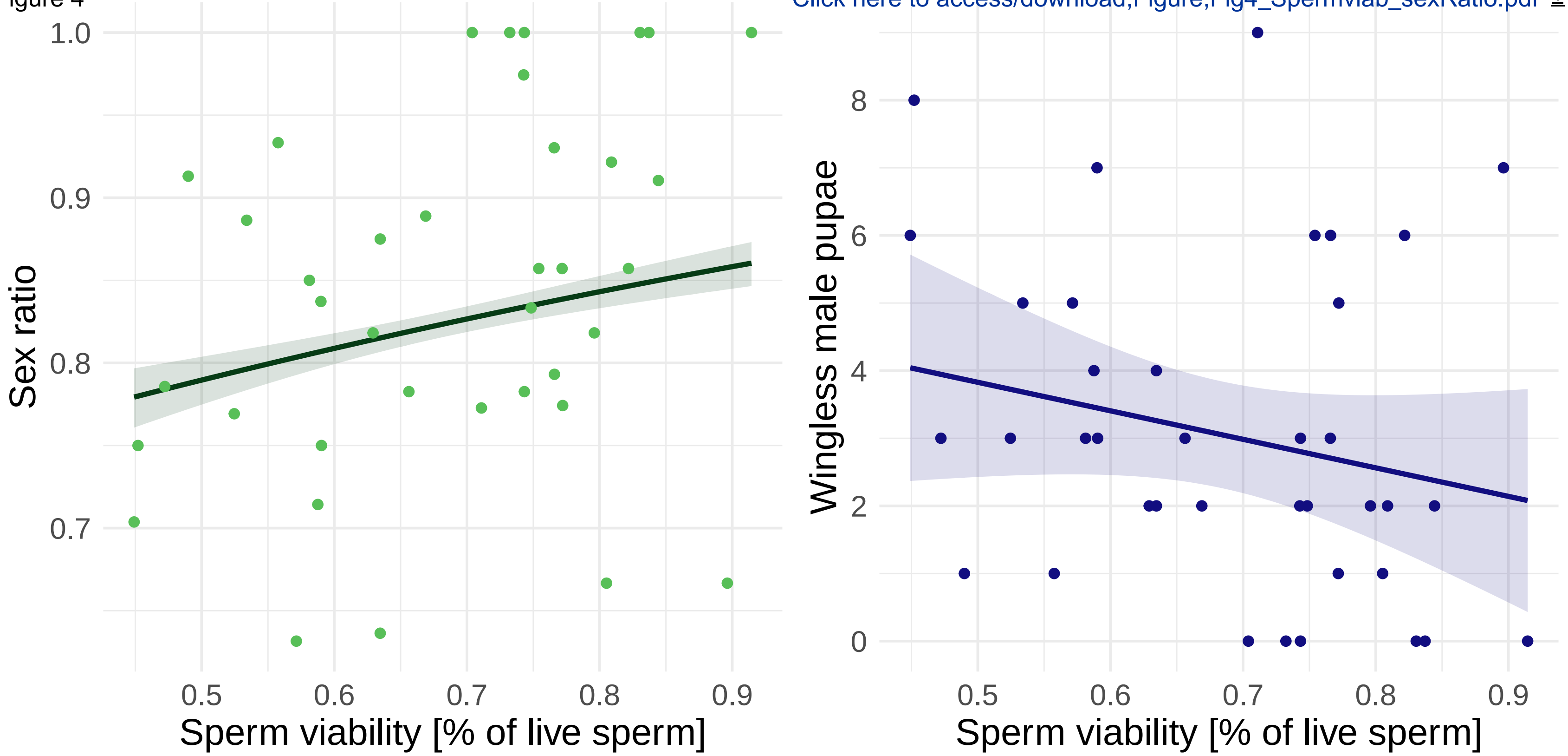

Supporting Information 1. Supplementary Figures

Click here to access/download
Supporting Information
SupplFile1_Fertility_antqueens_250804_LJ.docx

Supporting Information 2. Raw data

Click here to access/download
Supporting Information
SF2_merged_onlysacrificed_offset_sexualproduction.xls
x

Supporting Information 3. R Script

Click here to access/download
Supporting Information
SF3_CODE for manuscript NEW.R
