# Supplementary material for: Fertility correlates with queen size and sperm quality in an ant
Source: PLoS One. 2025 Dec 10;20(12):e0336378. doi: 10.1371/journal.pone.0336378 (PMC12694860; doi:10.1371/journal.pone.0336378)
Supplement: Attachment — Submitted filename: Response_Reviewers_PO.docx [file pone.0336378.s006.docx]

Review Comments to the Author

Reviewer #1: General comment

This study investigates how morphological traits of ant queens (Cardiocondyla obscurior) and sperm characteristics of males influence queen fertility, sex ratio, and caste allocation. Using 57 colonies, the authors tracked reproductive output over 12 weeks following the start of egg laying. They show that larger queens produce more workers and winged males but not more queens, while sperm viability (but not sperm length) correlates with female-biased sex ratios. The study highlights interactions between maternal size, paternal sperm quality, and reproductive outcomes, with implications for sexual selection and caste allocation in ants.

The authors make a good job highlighting their research question, the experimental design is overall sound, the statistical analyses are properly implemented, and the results are reported clearly with easy-to-apprehend graphics.

Nonetheless, I have several concerns about the experimental design (and thus the results) that need to be addressed.

Major comments

Comment 1 - Observation period

The monitoring period (12 weeks) is relatively short compared to C. obscurior queen lifespan (~25 weeks). In the study the authors provide for the justification of 12-week productivity as a good proxy for lifetime productivity (reference 41 in the manuscript), the model is indeed statistically significant, but the R2 is low-medium, indicating partial correlation.

Response: Thanks for this comment, we are aware that at 15 weeks of adult life, the correlation of female sexual production and egg production is statistically significant but has a lower correlation than the total egg production (r = 0.37 vs 0.74). We now acknowledged more explicitly this limitation in the discussion section. We included that in this species biomass investment into workers and queens are highly correlated (r = 0.762, p < 0.001) in Kramer et al, PLOS One (2015), and in (r= 0. 691, p < 0.001) in Jaimes et al, eLife 2022, which account for a high level of variability across queens.

The Discussion now reads in L313 “While 12 weeks is a good predictor of lifetime egg production (43), the production of sexuals reaches a maximum at around 30 weeks (26). It is possible that larger queens produce more sexuals once the ergonomic phase of the colony is surpassed, i.e., the phase of colony growth with the main investment in the workforce. Still, lifetime egg and sexual offspring production are positively correlated [r=0.762, and r= 0.691 depending on the study (26,27)], but whether larger queens produce more female sexuals later in life remains to be determined.”

In addition, this study appears to have been stopped at 15 weeks (not 12), which is close, but should be clearly stated for transparency.

Response: We have now clarified in the Abstract and in the Material and Methods that the monitoring period corresponded to 12 weeks after the onset of egg laying, and how this corresponds up to 15 weeks given the variability in egg laying onset. L121 “The queen was monitored for 12 weeks following the onset of egg laying (median onset=12 days after pupal eclosion, sd ± 3.21). After this 12-week period, the queen was frozen at -20°C. The colony was then monitored weekly until all larvae had eclosed. Overall, the total tracking period extended up to 15 weeks after queen pupal emergence, since the onset of egg laying varied between 9 to 23 days after mating.”

In addition, the authors clearly state in the introduction (line 66) that productivity is highly variable between colonies in this species. Moreover, previous studies from the same research group showed that queens shift to the production of sexuals in late life, suggesting that lifetime productivity warrants monitoring over the entire lifespan of queens, because early-stage monitoring can difficulty be extrapolated. Together, this questions the use of 12-week productivity as a good proxy for lifetime producitivity. The authors **should more explicitly address this limitation** and provide rationale as to why the 12-week period was chosen, instead of a longer one such as mean queen lifespan or lifetime monitoring.

Response: We have addressed now this limitation and provide a rationale for chosing the monitoring up to 12 weeks after egg laying begins. Studies showed that there is a high positive correlation between lifetime egg production and sexual production, and we expect that larger females will produce more female sexuals late in life. Still, we acknowledge that this needs further verification. L332 now states “It is possible that larger queens produce more sexuals once the ergonomic phase of the colony is surpassed, i.e., the phase of colony growth with the main investment in the workforce. Still, lifetime egg and sexual offspring production are positively correlated [r=0.762, and r= 0.691 depending on the study (26,27)], but if larger queens will produce more female sexuals late in life needs further study.”

Comment 2 - Sperm count

The number of counted sperm is very low (line 255: ‘1149 spermatozoa from 39 wingless males’, which is ~30 counted spermatozoa per male). Sperm viability assay using microscopy are typically performed on several hundreds of spermatozoa per individual (see, for instance, den Boer et al., 2010 – Science; Garcia-Gonzaez & Simmons 2005 – Current Biology; Hunter & Birkhead 2002 – Current Biology) to provide robust estimates of sperm viability.

Response: Thank you for pointing out the method was not clear. For sperm viability all cell imaged were counted as stated L142 “all live (green) and dead (red) sperm cells of the 5 images were counted”, exploiting to the maximum the data. This new version of the manuscript includes in the results section in L294 “A total of 1149 spermatozoa were measured for sperm length, and 24155 sperm cells were checked for viability from 39 wingless males.” This corresponded to an average of 619 sperm cells per male. For social insects “at least 300 sperm cells” are usually measured (Boer, Baer & Boomsma,2010; den Boer, Sturup, Boomsma & Baer, 2015), and other studied insects have measured 500 sperm per sample (Garcia-Gonzaez & Simmons, 2005; Hunter & Birkhead, 2002).

In terms of sperm length measuring 10 sperm/male has been a standard. For example, see “Mean sperm values were obtained for each individual male by measuring 10 sperm/male for all ant species and bumblebee species” (Fitzpatrick & Baer, 2011). In our approach we measured 10 sperm cells in each of the 5 different images obtained per male.

In addition, sperm viability oscillates between ~0.5-0.9, which reflects a large variance as compared to other social Hymenoptera (see, for instance, den Boer et al., 2010 – Science, Degueldre and Aron, 2023 – Journal of Zoology). The authors rightfully propose that this may be a consequence of low postcopulatory sperm competition and lifelong spermatogenesis. However, the high variance may result from technical issues related to insufficient sperm count.

Response: It does not seem to be the case, please see below.

The authors should address why they chose to limit their analyses to this low number of sperm, and discuss whether the number of counted sperm in their study is sufficient for robust estimates. If possible, the authors should exploit their photographs to increase the number of counted sperm per male, providing more robust estimates of sperm viability. If not, they should justify their choice to restrict their viability analyses to only a few sperm cells and explicitly acknowledge this limitation in the Discussion.

Response: We have now made clear in the manuscript that the amount of sperm counted to estimate the cell viability corresponded to ~619 sperm cells per male (see above). We have some males from which a low level of sperm was retrieved but the estimate of cell viability and coefficient of variation is similar when including data on those males with lower number of cells in the images taken.

In L295 we now state “Sperm viability was 68.4% (cv=18.6%) on average, which was similar to the average viability when considering only males with > 300 counted sperm cells (mean = 68.8%, cv=18.4%, n=27).”

We have now expanded the discussion of high variability on sperm viability in the males compared to what is observed for other social insect species and placed our findings in a broader context in L389 “Sperm viability is predicted to be higher in species with more intense sperm competition. Evidence from several insect taxa support this hypothesis, as monandrous species tend to exhibit a lower proportion of live sperm (51). For example, in the seminal vesicles of the polyandrous species Formica truncorum, over 90% of the sperm were viable, compared to a median viability of 75% in the monandrous Dinoponera punctata (51). Sperm viability influences paternity success in polyandrous insect species (52). In C. obscurior, females mate with a single male, and male competition occurs predominantly before mating, suggesting a relaxed post-ejaculatory selection on sperm viability that could explain their lower sperm variability (~68%). However, monandrous species not only show lower average sperm viability but also greater variability in sperm quality (51,53), also suggesting lower post-ejaculatory selection. An essay in the polyandrous species Acromyrmex echinator and Atta colombica reported 60-90% relative sperm viability, along with low variability among males (53). Exception to this pattern are the monandrous ants Trachymyrmex cf. zeteki and Temnothorax crassispinus, which both exhibited high sperm viability and low variability (53–55), warranting further investigation. Comparison among different studies is difficult given the differences in sperm and seminal fluid across ejaculates, and the different preservation media used.”

Comment 3 - Broader evolutionary context

The authors make a good job comparing their result to others in the discussion, however they are mostly limited to their species with a few exceptions. Further contrasting the results from this study in a broader evolutionary context in the final paragraph would add valuable perspective on the generality of the findings and their implications for understanding reproductive strategies across taxa.

Response: Thanks for pointing this out. As mentioned above, we have now contrasted our findings to those on other ant species where we felt relevant, with what we believe we have improved the relevance of this study.

Minor Revisions

Lines 52-54: the authors cite articles as reference for the existence of spermiophagy, yet these sources merely speculate that it may happen in the spermatheca, without providing direct evidence. I suggest formulating this sentence with more nuance to the authors’ claim.

Response: Thanks for pointing this out, we have modified the claim in L57 as “Further, spermiophagy during long-term sperm storage in ants could possibly eliminate dead sperm cells (12–15), which are a potential source of toxic molecules, enabling a more efficient fertilization of eggs, as suggested for *Melipona* bees (16).”

Lines 73-74: The use of ‘paternal body size’ is confusing. Do the authors mean ‘parental’?

Response: Exactly in this point we referred to “parental” we have corrected it.

Lines 75-76: The positive correlation between male body size and sperm quality should be more explicitly explained.

Response: We discuss the correlation between male size and ejaculates traits more broadly top give more context about this species, and what is generally observed for arthropods and vertebrates regarding male traits. Now the Discussion in L420 states “Paternal trait correlations showed that larger wingless males had more viable sperm. It is expected that costly sexual traits, such as sperm viability, are less expressed when individuals are in poor condition. Larger males, with a good nutritional status, might be better equipped to maintain the integrity of the germline. In several arthropods and vertebrates, body size responds stronger to changes in nutrition than sperm and ejaculate traits (6). In line with this, body weight of leaf-cutter ant males weight correlates with colony conditions (56). Males are on average heavier than virgin queens at the beginning of the pupal stage, but lighter at maturity (56), when spermatogenesis is arrested. This could reflect differential feeding rates after eclosion, but direct effects on sperm traits are unknown. Male body size in *Temnothorax crassispinus* show no correlation to sperm viability (55). Here instead, wingless male sperm viability positively correlated to head and thorax size, and these with mandible length, used to immobilize rivals during combat.”

With these revisions, the study would make a valuable contribution to understanding the correlation between inter-individual variations in parental traits and fitness in social insects.

Response: Thanks for the careful revisions, we have integrated them thoroughly.

Reviewer #2: General comments

The manuscript titled “Fertility correlates with queen size and sperm quality in an ant” (PONE-D-25-42359) addresses a relevant topic and makes a significant contribution to the field. In my assessment, the text is written in appropriate English and contains original results obtained from well-conducted experiments and analyses. The data support a coherent and pertinent discussion.

Response: Thanks for the careful revisions, we have integrated them thoroughly.

Regarding bibliographic references, I recommend that authors omit the months of publication and standardise the format by inserting appropriate spaces between citations. For example, instead of April 1, 1998;42(4):239–46, use 1998; 42(4): 239–46.

Response: We have kept the month in the references as this goes in line with the Vancouver citation format, outlines in the PlosONE guideline: https://journals.plos.org/plosone/s/submission-guidelines#loc-references

Therefore, I recommend accepting the manuscript for publication, but I suggest that the authors carefully evaluate the various suggestions—especially grammatical corrections—indicated in the attached manuscript file.
